# Examination of crime and similar concepts in the medical law

**Published:** 2016-05-01

**Authors:** Mohammad Javad Fathi

**Affiliations:** ^1^ Associate Professor, Farabi School, University of Tehran, Qom, Iran

**Keywords:** *Criminal conduct*, *Civil crime*, *Disciplinary transgression*, *Guilt*, *Criminal responsibility*, *Civil responsibility*

## Abstract

Crime is a human behavior that has captivated the thoughts of scholars of various disciplines throughout history. Philosophers, sociologists, psychologists and others have investigated and analyzed the concept of crime from different aspects. Crime is the main topic of criminal law, and in its legal meaning is a well-known term with a certain conceptual load that should not be confused with similar concepts such as guilt, civil crime (quasi tort), and particularly, the disciplinary transgression. Although crime has common points with all the above, it is an independent concept with unique effects, features, and descriptions that distinguish it from similar acts. This article aims to determine the difference between the concepts of crime, guilt, civil crime and disciplinary transgression through enumeration of the related issues as well as examples of medical disciplinary crimes and transgressions. Identifying and distinguishing these concepts can improve the procedure of prosecuting crimes and disciplinary transgression, bring punishment to criminals and transgressors, and facilitate compensation of pecuniary and non-pecuniary losses due to committers’ fault or failure. Thus we may avoid taking a wrong route that can lead to infringement of individuals’ rights.

## Introduction

Criminal law is a branch of public law that attempts to prevent crime by defining the acts or omissions that lead to public disorders and by specifying legal reactions for each of those acts. Moreover, if a crime is committed, public law will specify the committer’s extent of liability and the manner of selecting and prosecuting these reactions in the light of the real personality of the committers. Finally, public law will determine the appropriate means of correction for the committers (1).

All branches of law - including criminal law - are used as means of social control, and their goal is to organize the behaviors and activities of citizens. 

Nevertheless, the fundamental difference between criminal law and the other branches of law is that the former uses suppressing punishments against those who breach its commands. Criminal law tries to reflect the principal values that demonstrate the human way of social life, and subsequently uses punishment as a means of consolidating these values and assuring their observance. Accordingly, criminal law not only intends to support individuals, but also seeks to support the social structure (2).

As a rule, criminal law uses coarse means and suppressing punishments. Therefore, human societies employ this branch of law as the last resort to maintain social control and public order, but since “easier comes first”, they try to avoid criminalization and punishment when other methods are available (3). It is thus essential to identify crime and its features and attributes, and distinguish it from other concepts such as civil crime, guilt, and disciplinary transgression. Additionally, recognizing the common and differentiating features of the above-mentioned concepts will help prevent confusion of the term “crime” with other similar terms. 

In medicine, examination of crime and concepts such as civil crime and disciplinary transgression will certainly yield important results. Examples of medical crimes include abortion and violation of professional secrecy, which can be subject to public prosecution in criminal courts. Some instances of medical disciplinary transgressions would be deeds contrary to medical codes of conduct and imposition of unnecessary costs on clients, and are prosecutable only in medical prosecutors’ offices and courts. The latter are syndicate institutions and have been established to maintain the dignity and vocational respect of medical communities. One of the objectives of the present article is to clarify the concept and conditions of civil responsibility and the mode of making restitution, which should be claimed in the criminal and otherwise the legal court following the criminal conduct. Clearly, different authorities address crimes, disciplinary transgressions and restitution claims, and therefore lack of differentiation between the foregoing legal authorities will lead to initiation of claims to an incompetent authority. The obvious outcome will be prolongation of proceedings, loss of evidence, and finally, infringement of the beneficiaries' rights. 

**Part One: Definition of Crime**

Crime^1^ has been defined in different social and humanistic sciences as "behavior against order", "behavior against public feelings and emotions", and "behavior incongruent with social conscience and common sense". Crime is a human behavior and a social phenomenon that can be studied through different viewpoints. Philosophers, speculative theologians, anthropologists, sociologists, psychologists, biologists, statistics scholars, etc. have investigated crime from various stances. 

From the sociological viewpoint, crime (criminal conduct) is human behavior that is incongruent with the common norms and values of a society. According to Emile Durkheim, a certain conduct is considered a crime when it hurts the strong and obvious feelings of the public conscience. This means that it is the judgment made by the society that criminalizes a conduct, not its tangible features. Consequently, a conduct might (not) be considered a crime according to time and place. Based on this viewpoint, a crime is quite dangerous, that is, it is so "intolerable" that it can hurt "the strong and obvious feelings of the collective conscience", and the disruption that this conduct brings about in life is such that the need for its stoppage is felt. The reason for the intolerability of a crime is that it hurts a social group's values and norms, which have been ordered according to their beliefs (4).

It should be noted that "social values" are among the main topics of sociology. The term is attributed to those facts and issues that satisfy the society members' physical and mental needs, including values such as power, knowledge, wealth, respect, health, skills, affection, piety, etc. Accordingly, behaviors that are against these values are considered abnormal and unacceptable (5).

In law, crime is a phenomenon that can be studied both in its concrete and validity dimensions. A concrete study of crime is performed to discover the causes, conditions, and underlying factors of its occurrence. This is a practice of criminology. In criminology, the term “crime” is basically attributed to all antisocial conducts and tensions that hurt the society, and are caused by either psychological or social factors. In this domain, it is not very important if such conducts are subject to criminal law or are covered by the legal definition of crime. Here, the dangerous state of an individual is deemed as a sign of antisocial behavior and an illness, and so a treatment through protective measures is suggested (6).

^1^ Words that have been considered as equivalents and synonyms of the word *Jorm* in Farsi and Arabic include *Bezeh, Gonah, Ethm, Ma'siat, Khalaf, Khata, Taqsir, and Takhallof*. In English, terms such as violation, fault, wrong, sin, delinquency, and misdemeanor have been adopted.

In criminal law, the validity and mental dimensions of crime are studied, which is important because the negative aspects of law breaking behaviors are assessed in the light of the principles, rules, and values prevalent in the society. For each behavior, a certain legal sanction is specified which is proportionate to the danger that threatens individuals' and the society's rights. 

In the 19^th^ century, German and French schools attempted to offer a definition for crime. Von Listz and other proponents of the German school defined crime as merely "conduct that is against criminal law" and disregarded the mental element. Therefore, according to the German school, presence of the physical element of committing an unlawful act or omission, as well as the legal element (being contrary to the criminal regulations) are enough for realization of a crime. The French school, however, added the mental element to the constituents of crime, and consequently defined crime as “the wrongful manifestation of one’s will for which a punishment, in its specific meaning, has been determined according to the law” (7).

Article 2 of the Islamic penal code, enacted in 2013, defines crime by its two constituting elements and stipulates, “Any behavior, either an unlawful act or omission, for which a punishment has been specified by the law, is considered a crime”. It is clear that the legislator has considered both physical and legal elements necessary for realization of a crime, and has not addressed the mental element. Articles 144 and 145, however, state that the mental element is essential for realization of both intentional and unintentional crimes: “For realization of intentional crimes, not only should the committer be aware of the crime, but also his/her intention of committing the criminal act should be ascertained. In cases where realization of a crime is legally dependent upon its outcome, the committer’s intention of generating that particular result or awareness of its fulfillment should also be ascertained” (Article 144).

“Realization of unintentional crimes is dependent upon ascertaining the committer’s fault … including imprudence and inadvertency” (Article 145).

In consideration of Articles 144 and 145, the legislator should have included the mental element in the definition of crime in Article 2, and accordingly, he should have defined crime as “any behavior, either an unlawful act or omission, for which a punishment has been specified by the law and is attributable to its committer”.

In medicine crimes include intentional abortion (Articles 622, 623, and 624 of the Islamic penal code, enacted in 1996); disclosure of clients’ confidential information (Article 648 of the Islamic penal code, enacted in 1996); issuance of false certificates (Article 539 of the Islamic penal code, enacted in 1996, and Article 5 of the Law on Providing a Physician’s Certificate before Marriage, enacted in 1938); deceiving the clients (Article 5 of the Law on Medical, Medicinal, and Food and Drink Regulations, enacted in 1955, and Article 4 of the Law on Prevention of Sexual and Infectious Diseases, enacted in 1941); and refusal to help the injured (the Single Article on the Punishment of Refusal to Help the Injured and Abatement of Imminent Dangers, enacted in 1975). 

**Part Two: Features and Attributes of Crime**

In order to identify crime, one needs to recognize its features, which are listed below: 

1. Crime is always the outer conduct of a person, which is realized either through an act or an omission. Therefore, having criminal intent is not considered a crime, and the mere intention to commit a crime is not punishable. Moreover, holding beliefs is not criminating, but spreading certain beliefs may be in some cases (8). Article 23 of the Constitution of the Islamic Republic of Iran stipulates, “Inquisition is prohibited, and no one can be persecuted or reprimanded merely due to their beliefs”. 

2. Mere prohibition of an act does not denote it is a crime, while presence of punishment does. From a legal viewpoint, a prohibited act for which no punishment has been provided is not considered a crime even if it is evidently against public ethics and order of the society (9).

3. Committing a prohibited act for which the legislator has assigned punishment is a crime only if the committer has perpetrated it without an enabling cause and in a way contrary to what is his right. Therefore, committing an act that is legally banned in order to exercise one’s rights or to enforce legal regulations is not considered a crime. For example, if abortion is done with legal authorization, or if a person defends himself, his honor, his property, or his or another’s body, in the presence of all the stipulated conditions of Article 156 of the Islamic penal law^1^, the foregoing act is not considered a crime (10).

1 Article 156 of the foregoing law stipulates, “If a person commits a legally criminal behavior while observing the proper stages to defend himself, his honor, his female family members, his property, or his or another’s body against any present or imminent danger or aggression, in case the following conditions are met, he will not be punished:

A. The committed behavior is necessary to avert aggression or danger. 

B. The defense is based on logical indications or logical fear. 

C. The danger and aggression were not caused by the intentional conduct or aggression of the committer himself and the defense of the other person.

D. Immediate use of state forces is not possible or their intervention is not effective on the abatement of the aggression and danger.

4. Providing instruments of crime or conducts and operations that are only criminal attempts and are not directly related to the criminal act, are not considered crimes unless in specific cases where the legislator has explicitly criminalized them (11). In Article 664 of the Islamic penal code, the legislator has considered the attempt to make or change a key to be used in a crime as a crime by itself, and has specified 3 months to one year imprisonment and up to 74 whip strokes for the committer. 

5. The criminal act should be recognized as a crime by legally competent authorities and the legal formalities should be performed and enacted. According to the principle of the legality of punishments, no conduct is a crime unless it has been recognized by the law as such, and no punishment is executable and applicable unless it has been equalized to a criminal act by the law (7).

**Part Three: Distinguishing Crime from other Prohibited Acts**

With regard to the fact that after the Islamic Revolution of Iran, the legislator has tended to follow the Islamic penal law and apply its regulations, three sets of prohibited behaviors are identifiable in the Iranian legal system that need to be distinguished from crime. These behaviors include sin and guilt, civil crime or quasi tort, and disciplinary and administrative transgression, and are explicated in some detail below. 

   *A. Sin and guilt*

Scholars of the Islamic penal law have defined sin as an act that breaches the orders and prohibitions of Quran and Sunnah, or conduct that leads to the corruption of individuals or the society (12). Therefore, there is a certain punishment for anyone who disobeys the divine orders and prohibitions that will afflict the committer in this world and must be executed by Imam or the ruler and the judge appointed by him. Punishment may be in the form of a religious obligation (an atonement for sin) that the committer performs to cover and make his sin disappear, or a torment that will affect the committer in the hereafter, unless he shows sincere repentance and compensates the victim(s) for their loss and infringed rights (13).

According to Islamic law, if punishing the wrongdoer is beneficial for the wellbeing of the public, that is, if it is necessary for the abatement of corruption or preservation of people’s health, it is a divine right that cannot be disregarded. Based on the type of the infringed right, crimes may involve violation of either God’s right or man’s right, and are categorized accordingly. Moreover, based on considerations related to public interests, crimes are divided into crimes against religion (e.g. apostasy), crimes against someone’s life (e.g. murder), crimes against wisdom (e.g. drinking), crimes against property (e.g. theft), crimes against progeny (e.g. adultery and sodomy), crimes against honor and dignity (e.g. imputation of unchaste individuals), and crimes against public security and comfort (e.g. Muharabah) (14). 

Therefore, crime differs from sin, which is an issue of predetermined and discretionary punishment. The relationship between these two concepts is that of generality and peculiarity in some way, that is, sin includes any form of immorality, even if it is personal and the outcome affects the committer himself, for instance drinking, lying, and failure to perform one’s religious duties. Crime, however, is an issue of conventional criminal law and does not include sins in the above-mentioned sense (12). On the other hand, crimes such as violations of registration laws and customs regulations are not regarded as sin unless they infringe the rights of people (3).

Moreover, crime and sin are different with regard to their origins. Islamic criminal law is based on divine revelation and follows Quran and Sunnah. The origin of criminal law, however, is conventional and substantive, and is developed in each country according to the opinions of a group of its scholars and is enacted by the legislative body of that country (13). 

   *B. Civil crime*

In European law, civil crime refers to conduct leading to social liability and includes any act that harms others, and a person who commits civil crime is legally bound to recompense the inflicted damage. Indeed, social liability is a kind of tortuous liability and is a form of legal responsibility not conditional to contract, such as usurpation, destruction, causation, and vindication (15). 

The present discussion only applies to destruction and causation. According to Article 328 of the Social Law of the Islamic Republic of Iran, “Anyone who destructs other’s property is responsible for it and should return its equivalent or price, regardless of whether he has destructed it intentionally or unintentionally, and whether it is corpus or benefit …”. In addition, according to Article 331 of Social Law, “Anyone who causes the destruction of a property should return its equivalent or price …”.

Although punishable offence and civil crime are similar in some aspects, they are different in others, as can be seen below:

1. Punishable crime is a violation of a part of the law. For example, without a certain law, suicide cannot be considered a crime and the suicide accomplice cannot be punished. Nonetheless, civil crime can be inferred from any tort or imprudence that causes damage, needless of any certain law (10).
2. Punishable crime is realizable independently and without infliction of any damage (e.g. vagrancy or abortion), while civil crime is realizable only due to the damage caused to someone. Therefore, civil crime is inconceivable without damage infliction (8).
3. In punishable crime, the mental element and presence of evil and criminal intent is the realization condition, while civil crime is based on a tort (16).
4. The sanction for punishable crime is penalty, while for civil crime it is restitution and compensation for the damage in the form of returning the destructed property to its previous state or paying its equivalent or price (17).
5. Criminalization of punishable crime seeks to maintain public order, while civil crime is banned so as to support individuals’ personal interests and benefits.
6. Any person might commit civil crimes, even the minor or the insane, but such individuals will not be held legally responsible if they do.

It should be noted that the logical relationship between civil crime and punishable crime is generality and peculiarity in some way. Therefore, an act might be a punishable crime and yet, it may entail civil responsibility. For example, inadvertence in surgery that leads to amputation will entail criminal charges, while the perpetrator will have to recompense the damage inflicted on the injured party as well. 

On the other hand, there are some punishable crimes that do not lead to civil responsibility, such as beggary, vagrancy and abortion. Moreover, in some cases, an act might lead to civil responsibility but not be punishable by law, such as refraining from accomplishing an undertaking, usurpation, destruction, and causation. 

At any rate, if a certain conduct is the source of both criminal and civil responsibility, then the public action initiator will be the prosecutor, and the private action initiator will be the injured party or his inheritors. Additionally, the public action adjudicating body will exclusively be the criminal court of law, while in private actions, the adjudicating body can be the criminal or legal court of law based on the injured plaintiff’s request. In the latter case, if the private action is initiated in a legal court, then the court should wait for the outcome of the public action in the criminal court of law (18). 

   *C. Disciplinary Transgression *

There are different syndicates and factions in every society for professionals and laborers of the same occupation. These entities receive certain regulations so that they can do their job in a healthy and honorable way and preserve their existence and professional dignity. Therefore, if members of a syndicate commit a prohibited act related to their professional and administrative status, they will be sentenced to disciplinary punishment by the very syndicate (19). The Iranian medical community is an entity that applies disciplinary measures against those members who neglect their dignity and professional integrity. 

The disciplinary bylaw of addressing the syndicate and professional transgressions of medical and related practitioners enacted by the State Expediency Council on November 6^th^, 2004, has enumerated the syndicate and professional transgressions of the following practitioners in various articles: physicians, dentists, pharmacists; specialists and doctors of laboratory sciences, medical diagnostics, gynecology; practitioners of laboratory sciences, optometry, eudiometry, speech therapy, immunology, medical biotechnology, radiology, bio-radiology, radiation therapy, nursing, operating room, anesthesia, medicinal sciences, nutrition, disease control and prevention, oral and dental health care, health, rehabilitation, physiotherapy, medical biochemistry, social services and social care, clinical psychology, clinical psychology and gifted children, basic medical sciences, chiropractic, medical genetics, and other related branches of medical sciences. The respective disciplinary transgressions include: inadvertence in performing legal duties (subject of Article 3); disclosure of clients’ confidential information and type of disease (subject of Article 4); deeds contrary to medical codes of conduct (subject of Article 6); imposition of unnecessary costs on clients (subject of Article 7); alarming the client through unreal explanations of disease seriousness (subject of Article 8); prescription of addictive and psychotropic substances in unnecessary situations (subject of Article 9); non-observance of medical services tariffs (subject of Article 10); receiving illegal monies (subject of Article 11); seeking and directing clients from public institutes to private doctors’ offices (subject of Article 13); absorbing clients through deceiving advertisements (subject of Articles 14 and 15); using untrue titles (subject of Article 16); prescribing medicine without scientific justification (subject of Article 17); refusal to continue the treatment process (subject of Article 18); selling medicine, makeup, hygienic products and medical equipment in the doctor’s office without a license from the Ministry of Health (subject of Article 20); issuing prescriptions that are not based on scientific principles (subject of Articles 21 and 22); non-observance of size and other features of prescription forms and signs (subject of Article 24); employing incompetent individuals in the medical practice (subject of Article 25); failure to inform the Medical Council about the address of the medical practice (subject of Article 26); failure to extend the professional license after expiration (subject of Article 29); employing individuals who are not licensed to work in the doctor’s office (subject of Article 30); embarking upon treatments out of one’s field of expertise (subject of Article 31); embarking upon complementary medicine not consistent with previous treatments (subject of Article 33); failure to obtain the necessary licenses by overseas graduates before starting medical practice (subject of Article 32); therapeutic practice by medical professionals who have psychological, mental, or physical problems, out of the limits of their legal license (subject of Article 34); any form of advertisement without written permission from the Medical Council (subject of Article 35). 

Punishable crime is different from disciplinary transgression in some ways, as can be seen below:

1. *Coverage:* In the case of crime, the coverage extends to all members of the society, and sometimes the punishment even passes geographical borders and considers lawbreakers abroad liable to the internal law, even if they are not citizens of that particular country (Articles 3, 4,5, 6, and 8 of the Islamic penal law have clarified the ambit of criminal law). Therefore, punishable crime applies to all citizens of a country, while disciplinary transgression is limited to a certain syndicate or community such as judges, physicists, lawyers, etc. (20).
2. *Legislation:* Punishable crime is determined, and has been prudently and transparently enumerated, by the legislator. Punishable crimes are equal for all people, while disciplinary transgressions are determined by organizational, administrative and syndicate regulations. The type and nature of disciplinary transgressions are different from syndicate to syndicate, and any conduct that violates the dignity of a syndicate or organization can be examined and punished according to disciplinary principles, even if it has not been specified in the respective bylaws or regulations (16). Article 6 of the Disciplinary Bylaw on Addressing the Syndicate and Professional Transgressions of the Practitioners of the Medical and Related Professions stipulates, “Performing procedures contrary to medical codes of conduct by practitioners of the medical and related professions is prohibited, and these individuals should refrain from committing deeds that dishonor the medical community. Instances of such conduct are determined by medical ethics committees”. Moreover, Clause 1, Article 28 of the Medical Council Establishment Law enacted on November 6^th^, 2004 by State Expediency Council stipulates, “Non-observance of religious and legal principles or syndicate and professional regulations, and inadvertence in performing legal duties by practitioners of medical and related professions is considered as transgression, and transgressors will be sentenced to the following punishments in a case-by-case manner based on the intensity of the committed conduct and its plurality and repetition …”. As can be seen, syndicate transgressions have not been specified explicitly and are legislated by broad and general regulations.
3. *Goals:* The goal of criminology is to maintain public order and support the totality of the society. In disciplinary transgression, however, communities and syndicates attempt to preserve administrative and organizational order as well as the professional dignity of their members.
4. *Evidence of guilt:* In criminal courts, confession, clear signs, judge’s knowledge, and similar forms of substantiation are considered evidence of guilt, and some crimes are provable through a specific number of such evidence. Disciplinary courts, however, are not limited to legal evidence and can use proof and presumptions that may not have probative value in punishable crimes in order to support their decisions.
5. *Competent court:* Punishable crimes are investigated by competent judicial authorities such as criminal courts and prosecutors’ offices. Disciplinary transgressions, on the other hand, are addressed by disciplinary courts and offices that are run by members of the same syndicate. Article 28 of the Medical Council Establishment Law stipulates, “In order to investigate syndicate and professional transgressions of practitioners of the medical and related professions, the Medical Council will investigate cases of major medical disciplinary offense in the capital city, lower and court of appeals disciplinary transgressions in the capital cities of provinces, and minor medical disciplinary transgressions in the cities …”. Article 29 of this law discusses members of the prosecutor’s office, Article 35 specifies lower disciplinary transgressions, and Article 36 determines court of appeals disciplinary transgressions.
6. *Sanctions:* Sanctions against crime are punishments, which in the case of punishable crimes are provided by the law. In disciplinary transgressions, however, sanctions are disciplinary and administrative, for instance reprimand, deduction of wages, demotion, dismissal, etc. Therefore, disciplinary sanctions only affect the professional status of the committer and are lighter compared to criminal punishments, which can affect the criminal’s life (e.g. death penalty), body (e.g. whipping), freedom (e.g. imprisonment), property (e.g. fines and forfeiture), and dignity (e.g. public announcement of the decision) (21).

Clause 1, Article 28 of the Medical Council Establishment Law has enumerated the following disciplinary punishments:

- Oral warning or reprimand in the presence of the executive board of the respective Medical Council.
- Written warning or reprimand, to be filed in the respective Medical Council records.
- Written reprimand filed in the respective Medical Council records and published in the council press or installed on the respective Medical Council signboard.
- Deprivation from practicing medical and related professions in the location of transgression for duration of 3 months to one year.
- Deprivation from practicing medical professions for duration of 3 months to one year throughout the country.
- Deprivation from practicing medical professions for more than one year to five years throughout the country.
- Permanent deprivation from practicing medical and related professions throughout the country.

The logical relationship between crime and disciplinary transgression is also that of generality and peculiarity. For example, crimes such as embezzlement, bribery, abortion, issuance of false certificates, and disclosure of clients’ confidential information are considered disciplinary transgressions as well, while crimes such as theft or breach of trust are not considered disciplinary transgression. Moreover, disciplinary transgressions such as committing deeds contrary to medical codes of conduct, non-observance of the size and other features of the prescription form and sign, and failure to inform the Medical Council about the address of the medical practice do not have criminal aspects. 

It should be noted that a sentence issued in a disciplinary action does not affect criminal or civil action. Nevertheless, it is possible that a conduct is simultaneously the origin of disciplinary, criminal, and civil actions, such as a physician’s inadvertence in performing the legal duties leading to infliction of damages on the client, or prescription of medicine without scientific justification. In such cases, if the client suffers damages, in addition to disciplinary action, the physician will have criminal and civil liabilities as well. 

## Conclusion

Crime is the outer behavior of a person, which is sometimes realized as an unlawful act and sometimes as an omission, and the presence of punishment is a condition for its realization. The principle of the legality of punishments emphasizes criminalization of prohibited conduct and specification of punishments for transgressors and offenders by the legislator. This principle is used as the main criteria to distinguish crime from other similar concepts such as guilt, civil crime, and disciplinary transgression. Sin and guilt are noncompliance with divine laws and consequently have a divine origin based on revelation, and are inferred from Quran and Sunnah. Crimes, on the other hand, are conventional and substantive and are specified by the legislative body of each country. Moreover, civil crime is any conduct that inflicts damage on another person, and the committer is legally bound to recompense the inflicted damage. Therefore, civil crime might refer to any mistake or imprudence that causes damage, regardless of presence of a certain law. 

In addition, disciplinary transgression is a prohibited act that is related to the committer’s professional and administrative status and results in disciplinary action and punishment. Contrary to punishable crimes, which are specified by the legislator, disciplinary transgressions are determined through organizational and administrative bylaws and syndicate regulations. Furthermore, any conduct that jeopardizes the dignity of a syndicate or organization can result in disciplinary sentence and punishment, even if it has not been mentioned in the respective bylaws or regulations. 
